# Outcomes of Forest Landscape Restoration Shaped by Endogenous or Exogenous Actors and Institutions? A Systematic Review on Sub-Saharan Africa

**DOI:** 10.1007/s00267-023-01808-x

**Published:** 2023-03-21

**Authors:** Raphael Owusu, Jude Ndzifon Kimengsi, Lukas Giessen

**Affiliations:** 1 Forest Institutions and International Development (FIID) Research Group, Chair of Tropical and International Forestry, Faculty of Environmental Sciences, Technische Universität Dresden, Pienner Str. 7, 01737 Tharandt, Germany; 2 Department of Geography, The University of Bamenda, Bamenda, Cameroon; 3 Chair of Tropical and International Forestry, Faculty of Environmental Sciences, Technische Universität Dresden, Pienner Str. 7, 01737 Tharandt, Germany

**Keywords:** Actors, Endogenous, Exogenous, Forest landscape restoration, Forest policy, Institutions

## Abstract

Although ambitious, forest landscape restoration (FLR) is still very high on global climate change mitigation and adaptation research and policy agendas. The scientific literature highlights the importance of institutions and actors’ collaboration for achieving the intended outcomes. Despite these diffuse indications, a comprehensive understanding of the role played by different types of actors and institutions in shaping FLR outcomes is missing. This hinders the definition of an actor-cum-institutions research agenda for FLR, especially in sub-Saharan Africa (SSA). Yet, in this region, different actors with diverse interests shape FLR practices. Likewise, formal and informal institutions are known to collide frequently. Hence, this paper addresses the lacunae by systematically reviewing FLR actors’ interests and power manifestations and the typologies of institutions linked to FLR outcomes in SSA. The review further defines future research agendas on actors and institutions in SSA. The following lessons can be drawn from the review of 75 peer-reviewed journal articles: *First*, while exogenous actors are interested more in the ecological benefits of FLR, endogenous actors are interested in economic ones. *Second*, exogenous actors mostly use (dis-)incentives and coercion to shape the behavior of endogenous actors in FLR. *Finally*, while the exogenous formal institutional typology produces positive and negative ecological, economic, political, and sociocultural FLR outcomes, the endogenous formal and informal institutions produce only positive outcomes. Future studies should identify actors’ compliance levels of the exogenous and endogenous formal and informal typologies of institutions. Future studies should also analyze the effectiveness of FLR-linked institutions towards ensuring successful FLR.

## Introduction

Deforestation and forest degradation continue to ravage global forests at an alarming rate. About 10 million hectares of global forest area is deforested annually (FAO 2020). There is a worldwide interest in restoring degraded forests and landscapes to reverse the economic effects, mitigate climate change, and sustain the numerous ecosystem services that forests provide in the concept referred to as forest landscape restoration (FLR) (IUCN and WWF 2000; Stanturf and Mansourian 2020). Even though very ambitious, FLR — “the planned process that aims to regain the ecological integrity and enhance human wellbeing in deforested or degraded forest landscapes [and beyond]” (IUCN and WWF 2000:2) — in sub-Saharan Africa (SSA) is still very high on the international and national research and policy agendas in the bid to enhance climate change mitigation and adaptation, ensure food security and poverty alleviation (Pramova et al. 2012; Stanturf et al. 2015). The issue of governance is increasingly understood to be the main determining factor to the successes or otherwise in the environmental sector, especially the restoration of degraded forest landscapes (Carter et al. 2009; Guariguata and Brancalion 2014; Mansourian 2016).

The scientific literature generally highlights the importance of international to local actors’ collaboration across scales and policy sectors for achieving the intended effects and political pledges, such as the Bonn Challenge^1^ of 2011 and the AFR100^2^ (Mansourian 2017; Stanturf 2021). Several FLR interventions over the years have therefore adopted a multifaceted approach by rolling out interventions in degraded and deforested landscapes with the involvement of diverse actors with various roles, interests, and power potentials (Djenontin et al. 2021; Kiptot and Franzel 2012; Reij et al. 2020). For instance, in SSA, thousands of smallholder farming households with diverse interests, over the years, have tried to increase the number of trees on their croplands through deliberate tree-planting and by managing and protecting the trees that regenerate from seeds and rootstocks (Kiptot and Franzel 2012; Reij et al. 2020). Also, international and national level actors have collaborated with community-level actors in projects such as the Great Green Wall and Regreening Africa, geared towards reversing land degradation and desertification, improving food security and climate change adaptation (Mansourian and Berrahmouni 2021). In the same vein, the literature highlights the importance of institutions and the dynamics around institutions in different forest landscapes (Kimengsi et al. 2022a; Osei-Tutu et al. 2015; Yeboah-Assiamah et al. 2017).

Institutions are the structures (e.g., state agencies, chieftaincy, etc.) and processes (e.g., rules, norms, etc.) that shape human interactions by acting as constraints or enablement, especially in natural resource use and management (Fleetwood 2008; North 1990; Ostrom 1990). To Ostrom (1992), institutions are the set of rules-in-use by actors to organize repetitive activities that produce outcomes affecting those actors while potentially affecting others. They (institutions) are the outcome of political choices determined by either exogenous or endogenous entities (Shvetsova 2003). By implication, institutions could be either exogenous or endogenous. Endogenous institutions are the structures or processes designed by the people or socio-political structures that are likely to be affected by the designed structures or processes (institutions) (Shvetsova 2003). Exogenous institutions, on the other hand, are the control mechanisms introduced into a community by external entities (Vallino 2014). Based on their formality, the endogenous and exogenous institutions could be either formal or informal (Yeboah-Assiamah et al. 2017). The institutions in SSA have regulated the activities of diverse FLR actors in SSA: and have produced differential social, ecological, economic and political outcomes that are usually linked to the interest of some FLR actors (Asaaga et al. 2020; Folefack and Darr 2021; Laestadius et al. 2015; Walters et al. 2021). Moreover, the institutional arrangements triggered by specific actors’ interests also produce injustices in the local communities regarding who (does not) benefit from FLR (Elias et al. 2021; Kandel et al. 2021; Kariuki and Birner 2021). These may partly explain why FLR actors, on the one hand, and institutions, on the other hand, have attracted political and scientific attention, hence their recent growth in the literature from SSA.

Although the literature on FLR actors and institutions is growing, a comprehensive understanding of the role played by such different types of formal or informal, endogenous, or exogenous institutions, as well as types of actors in shaping FLR and the outcomes they produce is still missing. This hinders the definition of an actor-cum-institutions research agenda for FLR, especially in the context of SSA. Yet, in this region, actors from traditional local as well as post-colonial centralized realms shape FLR practices based on their diverse formal and informal interests. Likewise, formal and informal institutions stemming from endogenous local or exogenous national or international contexts are known to prevail and collide frequently. Hence, this paper addresses the lacunae by systematically reviewing (i) FLR actors’ interests and power manifestations; (ii) and the typologies of institutions linked to FLR outcomes in SSA. Based on these objectives, the review identifies future research priorities and questions regarding actors and institutions in SSA. It, therefore, defines future research agenda on FLR actors and institutions in SSA.

## Materials and Methods

### Analytical Framework

The analytical framework of this review paper hinges on two key concepts: exogenous and endogenous institutionalism (Shvetsova 2003; Vallino 2014) for analyzing the institutional dimension of the FLR literature; and the actor-centered power concept (ACP) (Krott et al. 2014) for addressing the actor dimension.

**Fig. 1:**
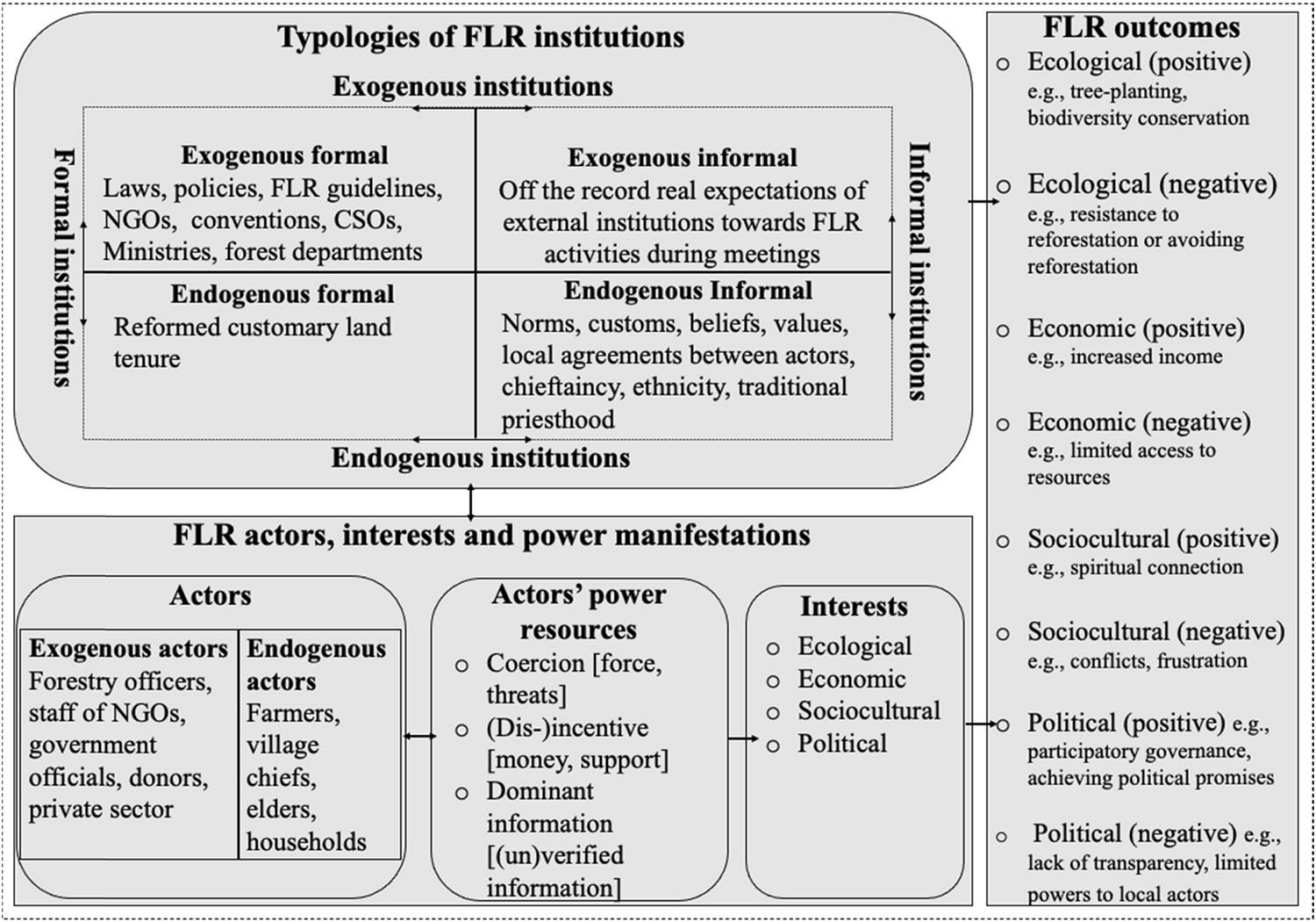
Analytical framework of the study [adapted from Kimengsi et al. (2021), Krott et al. (2014), Shvetsova (2003), Vallino (2014), Wibowo and Giessen (2015), and Yeboah-Assiamah et al. (2017)]

The exogenous and endogenous institutional analytical lens presents institutions as the outcome of external and/or local-level political choices that shape people’s behavior, especially in natural resources management and use (Shvetsova 2003; Vallino 2014). There are diverse levels of endogeneity and exogeneity. In this study, endogenous refers to the local communities where the various case studies were conducted. In contrast, anything outside the case study community is exogenous. Both institutions (exogenous and endogenous) have some formality; they are either formal or informal (Kimengsi et al. 2021; Yeboah-Assiamah et al. 2017). Formal institutions consist of written and codified processes and structures mostly driven by national governments: these include policies, laws, conventions, state agencies, etc. (Yeboah-Assiamah et al. 2017). Informal institutions, on the contrary, are the local levels’ unwritten or uncodified structures and processes, such as customs, taboos, chieftaincy, etc., that are mostly transferred from one generation to the other (Kimengsi et al. 2021; Yeboah-Assiamah et al. 2017). This, therefore, resulted in the four typologies of institutions, i.e., the exogenous formal^3^, the exogenous informal^4^, the endogenous formal^5^, and the endogenous informal institutions^6^ (Kimengsi and Mukong 2022; Kimengsi et al. 2022a) (Fig. 1). These typologies of institutions interact to moderate the behavior of resource users and managers (actors) (Yeboah-Assiamah et al. 2019), hence shaping the outcomes of the resources management (for this case, FLR) (Fig. 1).

The outcomes of FLR interventions could be (a) ecological (positive), e.g., tree-planting^7^, soil improvement, biodiversity conservation; (b) ecological (negative), e.g., resistance to reforestation or avoiding reforestation^8^; (c) economic (positive), e.g., increased income, increased fodder availability, improvement in local people’s livelihood; (d) economic (negative), e.g., market constraints, limited access to resources; (e) political (positive), e.g., participatory governance, achieving political promises or commitments; (f) political (negative), e.g., lack of transparency, limited powers to local actors; (g) sociocultural (positive), e.g., spiritual connection to the land, ancestral spirits and gods; (h) sociocultural (negative), e.g., conflicts, frustrations of rural poor people (Fig. 1). The actors also sometimes employ their power potential to shape the institutions to pursue their interests (Fig. 1).

The ACP approach posits actors as entities that have interests and the potential to influence processes and/or other actors in the bid to pursue their interests (Krott et al. 2014; Schusser et al. 2015). These actors could be exogenous^9^ or endogenous^10^: and their interests are an integral part of their decision-making architecture (Schusser et al. 2016). The interests of FLR actors are the factors that motivate them to participate or undertake FLR intervention willingly: these could be associated with the ecological, economic, sociocultural and/or political benefits of FLR (Gakou-Kakeu et al. 2022; Palmer et al. 2022; Sanou et al. 2019; Walters et al. 2021). In pursuing their interests, most actors alter the behavior of other actors without recognizing their will: and may as well create or modify institutions to help secure their interests (Krott et al. 2014; Wibowo and Giessen 2015). This process of shaping institutions and the behavior of other actors against their will, referred to as power, is mostly achieved through coercion (force), (dis-)incentive (money, support), and dominant information (unverified information) (Krott et al. 2014). In the context of FLR, the use of power by exogenous and endogenous actors (Fig. 1) to secure their interests linked to the ecological, economic, political, and sociocultural benefits of FLR is likely to affect FLR interventions and the overall outcomes of FLR.

### Method

#### Data collection

**Fig. 2:**
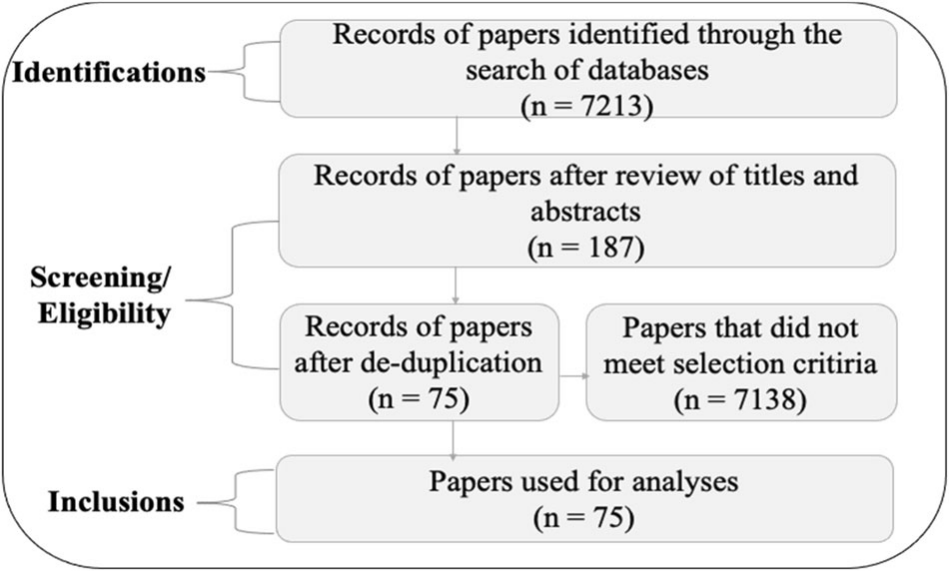
Systematic review flow of the number of papers [adopted from Kimengsi et al. (2022b), and Petticrew and Robert (2006)]

A systematic review approach was employed to collect relevant empirical and peer-reviewed journal articles on institutions and actors that shape FLR activities in SSA (Kimengsi et al. 2022b; Petticrew and Robert 2006). A list of search terms was developed to aid the literature search in the Science Direct, Web of Science, Scopus, and Google Scholar databases. First, the main themes of FLR (e.g., forest landscape restoration, tree-planting) were combined with themes of institutions (e.g., policies, taboos) and actors (e.g., forestry officials, community members): this was then combined with themes from SSA (e.g., Sub-Sahara Africa) (see supplementary file 1 for details). The literature search occurred between April and September 2022 and revealed a total of 7213 papers (Fig. 2); i.e., 1429 from Scopus, 740 from Google Scholar, 2505 from Science Direct and 2535 from Web of Science. This was reduced to 187 papers after reviewing the abstracts and in some situations, introductions, methods, results and conclusions (when abstracts did not provide detailed information to guide the determination of inclusion or exclusion). Inclusion was based on (a) papers published in English, (b) papers on FLR-linked institutions and actors in SSA, and (c) papers that are empirical and published in peer-reviewed journals. After de-duplication, the papers were reduced to 75 (Fig. 2). The 75 empirical and peer-reviewed journal articles reported results of 115 case studies on FLR-linked institutions and actors in SSA.

The retained papers were carefully reviewed by focusing on the variables or proxies in the analytical framework, i.e., typologies of institutions, FLR actors, actors’ interests, actors’ power resources, and FLR outcomes (Fig. 1). Microsoft (MS) Excel was used to create a database covering variables and other interest sub-variables (Artmann and Sartison 2018; Kimengsi et al. 2022b) (see supplementary file 2).

### Data Analyses

Directed content analysis was used to analyze the literature (Clay et al. 2015; Hsieh and Shannon 2005). Considering that the subjects of institutions as well as actors, their interests, and power manifestation are complex and sometimes not framed directly in studies, the use of software to extract data from studies on such complex subjects might miss out on certain relevant information (Kimengsi et al. 2022b). This informed the use of directed content analysis. We read the papers’ abstracts, introductions, methods, results and conclusions and extracted salient information linked to the subject of interest. The key information that directly or indirectly expresses one or more of the variables was recorded in the MS Excel spreadsheet by employing the deductive approach (Artmann and Sartison 2018; Kimengsi et al. 2022b). The MS Excel application aided in conducting descriptive statistics. We explore the characteristics of the papers and case studies on FLR institutions and actors in SSA. Also, the Spatio-temporal distributions of the typologies of FLR institutions and actors in SSA were obtained. Additionally, descriptive statistics aided in obtaining the FLR institutions and actors in SSA. We tabulated the various actors’ interests, the power resources, and the outcomes of FLR linked to the different typologies of institutions. A Single Factor Analysis of Variance (ANOVA) in MS Excel aided in determining whether or not the observed variations in (a) actors’ interests, (b) actors’ power resources, and (c) FLR outcomes were statistically significant. A mapping software, QGIS (version 3.22.7), was used to design the map of Africa that indicates the distribution of case studies on FLR-linked institutions and actors in SSA. Narratives were used to elaborate on the information from the descriptive statistics.

## Results and Discussion

### Characteristics of the Literature on FLR-Linked Institutions and Actors in SSA

#### Sub-regional dynamics

This review categorized the literature on FLR-linked institutions and actors into two eras to capture the literature within the first 11-year period (2000–2011) and the second 11-year period (2012–2022) after the introduction of the concept of FLR in the year 2000 (IUCN and WWF 2000).

The review showed that more than 77% of the empirical peer-reviewed papers on institutions and actors in FLR in the SSA region were published from 2012–2022 (Table 1). This renewed scientific interest in FLR-linked institutions and actors could be attributed to the Bonn Challenge in 2011, when the need to restore a substantial area of degraded and/or deforested landscapes was reignited, which also saw the multiplication of actors in FLR. The review indicated that about 34% of the papers on FLR-linked institutions and actors in SSA reported information from western Africa (Table 1). Eastern and southern Africa sub-regions followed in that order (recording, respectively, about 30 and 25% of the papers on FLR-linked institutions and actors in SSA), with central Africa recording the least proportion of papers (about 10%). The results could be so because, until recently, restoration was not more of a priority in Central Africa. Also, since Central Africa is a highly French-dominated region, there is a higher potential of having more papers in French, thereby accounting for why the region recorded the least number of papers in this English-based review. However, for western and eastern Africa, their dominance in the FLR-linked institutional studies in SSA could partly be linked to the proximity of these regions to the Saharan areas and the urgency to tackle the rapid spread of desertification in these areas. Additionally, considering that Ethiopia, the highest committing country of AFR100, is in eastern Africa, and many countries in western Africa have committed massively to the AFR100, this may explain why these two regions recorded the highest papers on FLR-linked institutions and actors in SSA.

**Table 1:** Spatio-temporal distribution of papers and case studies on FLR-linked institutions and actors in sub-Saharan Africa (SSA)

Sub-regions in SSA	Frequency (*f) and percentages (%)* of papers and case studies across years							
	*Papers*				*Cases*			
	*2000*–*2011*	*2012*–*2022*	*Total*		*2000*–*2011*	*2012*–*2022*	*Total*	
			*f*	*%*			*f*	*%*
Western	6	21	27	34.2	10	35	45	39.1
Central	2	6	8	10.1	5	8	13	11.3
Eastern	5	19	24	30.4	5	24	29	25.2
Southern	5	15	20	25.3	9	19	28	24.3
Total *f*	18	61	79	100	29	86	115	100
%	22.7	77.3	100	100	25.2	74.8	100	100

Some papers reported information from multiple sub-regions

Regarding case studies, similar to the peer-reviewed journal papers, 2012–2022 recorded more than 70% of the case studies on FLR-linked institutions and actors in SSA; western Africa recorded the highest number of case studies, followed by eastern, southern and central Africa in that order.

#### Country-level dynamics

**Fig. 3:**
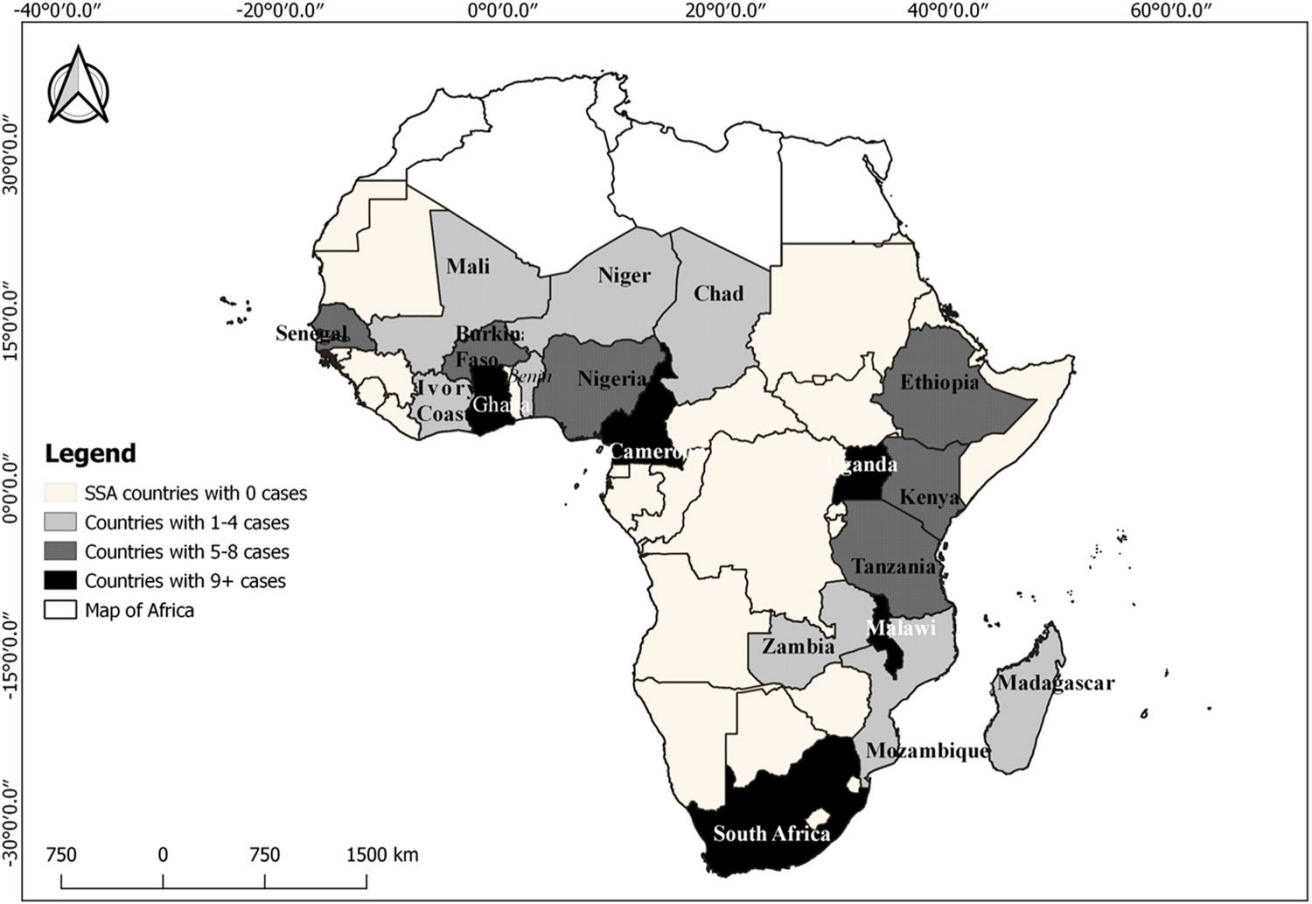
The map of Africa showing the distribution of case studies on FLR-linked institutions and actors across the sub-Saharan Africa (SSA) region

The 115 case studies used for this review emanate from 19 SSA countries (Fig. 3). The case studies on FLR-linked institutions and actors were unequally distributed, in both the inter-sub-regions and intra-sub-regions (Fig. 3). Concerning inter-sub-regions, western Africa recorded case studies from eight countries, five countries from southern Africa, four from eastern Africa, and two from central Africa. At the level of intra-sub-regions, only Ghana, in western Africa, recorded more than nine case studies (representing more than 7% of the total case studies in this review). In Central Africa, Cameroon recorded the most case studies (more than nine case studies) on FLR-linked institutions and actors. Uganda, in eastern Africa, recorded the highest number of case studies (more than nine cases). Three countries in the sub-region (Kenya, Tanzania, and Ethiopia) also recorded a significant number of case studies, between five and eight per country. In Southern Africa, Malawi and South Africa recorded significant numbers of cases studies (above nine) each.

### FLR Actors in Sub-Saharan Africa

The review showed that both exogenous and endogenous actors pursue FLR activities in SSA; however, the exogenous actors were the more dominant actors in FLR case studies in SSA (Table 2). Although at different levels, both exogenous and endogenous actors recorded more actors within 2011–2022 than the number of actors recorded in the literature published between 2000 and 2011. That is, while 106 exogenous actors and 88 endogenous actors were recorded within 2011–2022, 42 exogenous actors and 33 endogenous actors were recorded within 2000–2011. Concerning the sub-regional distribution, while eastern Africa recorded the highest and southern Africa had the least number of exogenous actors between 2000 and 2011, western Africa recorded the highest with Central the least number of exogenous actors within 2012–2022. The reason for these dynamics is unclear, especially considering that eastern and central Africa have countries with the highest commitment to the Bonn Challenge, yet these sub-regions did not record the highest number of actors between 2012 and 2022. On the other hand, the endogenous actors were recorded more from the literature from western Africa and least from central Africa in both periods, 2000–2011 and 2012–2022. An example of the role of exogenous actors in SSA’s FLR was reported by Walters et al. (2021), where the staff of an international donor agency (DGIS) provided resources to fund the livelihood and landscape strategy project that the government officials of Burkina Faso implemented from 2007 to 2010. Also, Foundjem-Tita et al. (2021) reported that more farmers in recent times and local environmental committee members (endogenous actors) in rural Chad maintain existing trees and plant new trees on their farms.

**Table 2:** Spatio-temporal distribution of FLR actors in sub-Saharan Africa (SSA)

Sub-regions in SSA	Frequency of FLR-linked actors in SSA across years					
	*Exogenous actors*			*Endogenous actors*		
	*2000*–*2011*	*2012*–*2022*	*Total*	*2000*–*2011*	*2012*–*2022*	*Total*
Western	12	39	51	12	36	48
Central	8	10	18	3	7	10
Eastern	16	32	48	10	29	39
Southern	6	25	31	8	19	28
Total	42	106	148	33	88	125

Table 3 presents the list of the exogenous and endogenous actors recorded in FLR-linked literature in SSA.

**Table 3:** Actors that engage or shape FLR activities in sub-Saharan Africa (SSA)

Sub-regions in SSA	Typologies of Actors	
	*Exogenous actors*	*Endogenous actors*
All sub-regions	Forestry officers; staffs of NGOs; government officials, donor agencies staffs; private sector	Local or commercial farmers; traditional leaders (village chiefs, elders); households
Western	Forest service division officers; regional & district plantation development officers; forestry commission officers; Staff of Great Green Wall agency	Taungya headmen; FMNR participants; assemblyperson; local forest management members; traditional priests
Central	Police officers; members of women organizations; environment officers; Mayors, REDD + council officers	Members of local environmental committees
Eastern	Members of productions & environmental committees; local council chairpersons; farm radio international representatives	Community based organization members; families; farmers associations; representatives of PFM groups
Southern	Provincial & district agricultural coordinators; agroforestry workers; Members of national FLR working group	Fuelwood sellers
Western, Central, Eastern	Representatives of IUCN	Forest harvesters
Western & Southern	Agricultural extension officers; CSOs representatives	Senior men from household
Eastern,Western	World Vision International staff	Landowners; tree growers
Western, Eastern, Southern		Community members

Source: based on Foundjem-Tita et al. (2021); Sanginga et al. (2010); Toth et al. (2019)*;* Walters et al. (2021); and others

#### Actors’ interests in FLR

The review showed that both exogenous and endogenous actors of FLR pursue FLR activities because of their interests in the potential ecological, economic, sociocultural and political benefits of FLR (Table 4). The review results (*F*_(3,12)_) = 2.948, *p* = 0.0758) and (*F*_(3,12)_ = 11,319 *p* = 0.0008) respectively show a statistical difference between the exogenous and endogenous actors’ interests in FLR. Across all the sub-regions of SSA, while the exogenous actors’ interests were more skewed towards the ecological benefits of FLR, the endogenous actors were more interested in the economic benefits of FLR than ecological, political and sociocultural. On average, more than 58% of the exogenous actors are interested in the ecological benefits of FLR, against 2%% for sociocultural benefits, whiles about 80% of the endogenous actors in FLR interventions in SSA are interested in economic benefits against 13, 6, and 5% in the ecological, sociocultural and political benefits. The results suggest that while exogenous FLR actors in SSA over these years may have placed more emphasis on the ecological outcomes of the FLR as against the other benefits, the endogenous actors are likely to prioritize the economic outcomes of FLR more than the other outcomes of FLR.

**Table 4:** Proportion of interests linked to the typologies of FLR actors in sub-Saharan Africa (SSA)

Typologies of Actors	*Sub-regions in SSA*	Actor’s interests in FLR				*F* value (*p* value)
		*Ecological*	*Economic*	*Sociocultural*	*Political*	
Exogenous actors	Western	30 (57.7)	18 (34.6)	0 (0.0)	4 (7.7)	3.373* (0.0546)
	Central	7 (70.0)	2 (20.0)	0 (0.0)	1 (10.0)	
	Eastern	10 (47.6)	10 (47.6)	0 (0.0)	1 (4.8)	
	Southern	7 (63.6)	3 (27.3)	1 (9.1)	0 (0.0)	
Endogenous actors	Western	5 (18.5)	21 (77.8)	1 (3.7)	0 (0.0)	11.319*** (0.0008)
	Central	0 (0.0)	7 (87.5)	0 (0.0)	1 (12.5)	
	Eastern	4 (24.3)	19 (79.2)	3 (11.5)	0 (0.0)	
	Southern	1 (8.3)	9 (75.0)	1 (8.3)	1 (8.3)	

[Numbers in parenthesis are the percentages of the actors in each sub-region whose interests in FLR are linked to ecological, economic, sociocultural or political; degree of freedom between group = 3; degree of freedom within group = 12]

Concerning the exogenous actors, the key ecological benefits of FLR that are of interest to exogenous FLR actors are reducing forest loss, reversing desertification, mitigating climate change, and enhancing carbon stocks. For example, in Cameroon, an FLR project that ran between 2009 and 2015, coordinated by forest and REDD + council officials, intended to plant trees to slow forest cover loss, enhance carbon stock, and stabilize slopes to prevent landslides (Gakou-Kakeu et al. 2022). The main economic benefits of interest to exogenous FLR actors are the enhancement of rural livelihoods, sustainable food production, and addressing limited fodder and feed supply. In Malawi, for instance, the Fodder Tree Technology initiative was a strategy by the Forestry Extension officers and government officials to address the limited fodder and feed supply in Malawi (Toth et al. 2017).

Also, the main sociocultural benefits of interest to exogenous FLR actors in SSA are a resilient social system and equity in access to ecosystem services. For example, in South Africa, the government officials implementing the Tsitsa landscape restoration project were interested in achieving resilient social and ecological systems by fostering equity in access to ecosystem services (Palmer et al. 2022). The political pledges on FLR and the increase of local ownership of the restoration activities are the main political issues of interest to the exogenous FLR actors in SSA. For instance, in Cameroon, the government’s policy to restore the landscape, coordinated by the forest and agricultural officers, is motivated by the political pledge to restore 12 million ha of the deforested or degraded landscape by 2030 (Mbile et al. 2019).

With respect to the endogenous FLR actors, the main economic interests of endogenous FLR actors are the generation of additional income, food and easy access to firewood. For example, in Niger, farmers showed interest in the farmer-managed natural regeneration program because the products of the baobab trees used for the restoration program provide food to the household and income by selling some of the products in the market (Agúndez et al. 2020). The key ecological interests of endogenous FLR actors are climate change mitigation, biodiversity conservation, and halting desertification. For instance, in northern Kenya, the motivation for local community members to participate in FLR activities was to halt the desertification process and restore productivity and biodiversity in pastoral drylands (Olukoye and Kinyamario 2009). The main sociocultural interests of endogenous FLR actors are social recognition and the cultural values of biodiversity. For instance, in Uganda, one of the main incentives that motivate community members to monitor a reserved area is to gain special recognition during social functions by the communities’ chiefs, elders and officials of the government (Acema et al. 2021). The key political interest of endogenous actors in FLR is land ownership rights. In Malawi, for example, most women who marry and reside in communities outside their native communities usually invest in agroforestry to legitimize their land ownership rights after purchasing a land (Benjamin et al. 2021).

#### Actors’ power resources in FLR

Power is the process of using elements or resources such as coercion (force), (dis-)incentive (money, support), and dominant information (unverified information) to change the behavior of actors against their will (Krott et al. 2014). The review revealed that exogenous actors in SSA use three different power resources (coercion, (dis-)incentive, and dominant information) to shape the behaviors of endogenous actors to pursue their interests in FLR (Table 5). There was, however, no information on the different power resources employed by the endogenous actors. With respect to the exogenous actors’ power resources recorded in the literature, the result (*F*_(2,9)_ = 10.697, *p* = 0.0042) indicates a significant difference between the rate at which the three power resources were employed by actors, with the use of (dis-)incentives being the most used power resource, followed by coercion and then dominant information sparingly used. At the sub-regional levels, while the (dis-)incentives as a power resource were mostly employed by the exogenous actors in western and eastern Africa, coercion and dominant information were mostly used by the exogenous actors in the southern and eastern Africa sub-regions (Table 5).

**Table 5:** Power resources used by FLR actors in sub-Saharan Africa (SSA)

Typologies of Actors	*Sub-regions in SSA*	Actor’s power resources			*F* values (*p* value)
		*Coercion*	*(Dis-)incentive*	*Dominant information*	
Exogenous actors	Western	7	41	5	9.738*** (0.0056)
	Central	4	15	1	
	Eastern	8	36	8	
	Southern	10	18	8	
Endogenous actors	Western	0	0	0	0.000 (NA)
	Central	0	0	0	
	Eastern	0	0	0	
	Southern	0	0	0	

[Degree of freedom between group = 2; Degree of freedom within group = 9; *NA* = not applicable]

Incentives as power resources in FLR in SSA include providing technical support or training, cash support, tree nurseries, tree seedlings, improved crop seeds and alternative income-generating activities such as beekeeping and small animal rearing. For instance, in Ghana, farmers that participated in the Community Resources Management Area restoration project received cash support, free saplings, and additional income generation activities like small animal rearing and beekeeping from the staff of an NGO and its funding partners (Walters et al. 2021). The use of disincentives by exogenous FLR actors as a power resource element is linked to the payment of fines. For instance, a community in Cameroon pay a fine of about US$ 400.00 for grazing their animals in a reserve forest (Ashley et al. 2006).

Coercion, on the other hand, is linked to using fences to protect or enclose an area, monitoring, policing, and arresting forest offenders. For example, local government officials in Uganda supported the restoration of degraded landscapes by policing the forest and monitoring and arresting forest offenders (Turyahabwe et al. 2007).

The use of dominant information in SSA’s FLR is about the use of public education and extension services to change how other actors of FLR behave towards landscape restoration in SSA. For instance, some farmers in the community resources management project in Ghana were encouraged that adopting an on-farm restoration focus would increase their productivity and diversify their incomes: this increased their participation in on-farm tree-planting (Baruah et al. 2016). In Uganda also, a radio program on FLR influenced about 98% of the community members who heard most of the broadcast to practice at least a restoration activity (Hampson et al. 2017).

### Typologies of Reported Institutions in FLR Studies

**Fig. 4:**
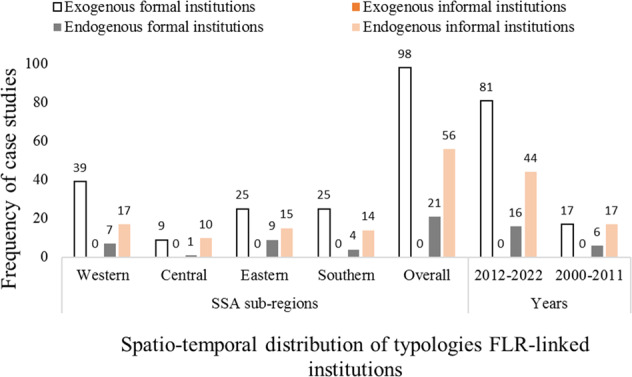
Distributions of the typologies of institutions observed to shape FLR activities in sub-Saharan Africa (SSA)

This review identified the typologies of institutions that shape FLR activities in SSA. Currently, information on three of the four proposed typologies of the institution (exogenous formal institution, endogenous formal institutions, endogenous informal institution) exists in the FLR-linked institutional literature in SSA (Fig. 4). The review did not obtain any information on the exogenous informal institutions in the FLR-linked institutional literature in SSA (Fig. 4). This could be attributed to the extent to which researchers find it very difficult to observe the unofficial agreements and expectations on FLR between exogenous entities and local communities.

Concerning the three typologies of institutions that are recorded in FLR-linked literature in SSA, while 98 of the case studies used for this review (about 85%) captured information on exogenous formal institutions, 21 case studies (about 18%) captured information on endogenous formal institutions and 56 case studies (about 49%) recorded information on the endogenous informal institutions that shape FLR activities in SSA (Fig. 4). The review shows that exogenous formal institutions are the most dominant typology of institutions in the FLR-linked institutional literature in SSA. This could be attributed to the possible difficulty in unraveling local-level and traditional structures and processes (endogenous formal and informal institutions) that shape FLR activities in SSA than to unravel exogenous formal institutional roles in SSA’s FLR. Nevertheless, the number of recent case studies that have reported on endogenous formal institutions has marginally increased (Fig. 4), suggesting a gradual research emphasis and efforts to unravel issues associated with this typology of institutions.

The typologies of institutions are unevenly distributed in the FLR-linked literature across the sub-regions in SSA (Fig. 4). While the western Africa dominated in the case studies that captured exogenous formal and endogenous informal institutions, followed by eastern and southern, the case studies from eastern Africa recorded the highest number of endogenous formal institutions, followed by western and Southern Africa (Fig. 4). The central Africa sub-region recorded the least number of case studies that reported on all three typologies of institutions, probably because this region recorded the least number of case studies that were analyzed for this review. Nevertheless, only central Africa captured more case studies on endogenous informal institutions than exogenous formal institutions (Fig. 4). This implies that the endogenous informal institutions might still be very influential in regulating FLR activities in the sub-region. In all, diverse institutions make up each of the three typologies of institutions that shape FLR activities in SSA. With respect to the exogenous formal and endogenous informal institutions, while some institutions are recorded in all the sub-regions, others are unique to specific sub-regions. On the other hand, the institutions that make up the endogenous formal institutional typology are unique to particular sub-regions in SSA. See Table 6 for details.

**Table 6:** Typologies of institutions that shape FLR in sub-Saharan Africa (SSA)

Sub-regions in SSA	Typologies of Institutions		
	*Exogenous formal institutions*	*Endogenous formal institutions*	*Endogenous informal institutions*
All sub-regions	Land tenure laws/policies, protected area policy, forests laws & policies, international & national environmental NGOs, ministries/departments of forest & environment, donor agencies		Customary (traditional authorities), customary land tenure arrangements, norms, customary rules
Western	National plantation development program & fund, forest & wildlife policies, modified taungya system, community resource management areas, national REDD + strategy, forestry commission, land allocation & taungya management committee, Great Green Wall program, small-scale forest restoration initiatives, forest management plan	Local forests management cooperatives, village forest management committees	Communities’ rules on forests, parkland agroforestry (norms)
Central	National REDD + steering committee, NTFPs trade law, national rural investment plan, action plan for UNFCCC, national tree planting week ordinance	Reformed customary land tenure structure	Women associations, farmers association, informal stakeholders’ agreement, sons and daughters’ association
Eastern	Community-based natural regeneration project, tree planting by-laws, shea tree management rules, PES, international small group tree planting program, forest conservation program, women’s promotion group, regional & district agricultural office, tree growers’ association, national environment management authority, national forest resources and agroforestry center, land rehabilitation programs	Village environment management committees, religious women’s group, cultural & religious organizations, community forest associations	Local rules on forest restoration
Southern	National strategy for FLR, forestry projects on ecological restoration & rehabilitation, district environment sub-committee, national FLR working group, tree for homes program	Marriages, catchment conservation committee, local forest management board	Ethnic groups, sacred sites, cultural practices
Southern, Western	Civil society organizations		
Eastern, Western	Forest management policy, collaborative resources management program, convention on biological diversity	Local government, youth associations	Forest users’ group
Western, Eastern, Southern	Agroforestry intervention		
Central, Western	Cocoa agroforestry & certification, REDD+		
Central, Eastern, Southern	AFR100, Bonn Challenge		
Eastern, Southern	World Agroforestry Center		

Source: based on Foundjem-Tita et al. (2021); Sanginga et al. (2010); Toth et al. (2019); Walters et al. (2021); and others

#### Typologies of reported institutions in FLR studies and their link to FLR outcomes

FLR institutions in SSA produce positive and negative ecological, economic, political, and sociocultural outcomes (Table 7). While the exogenous formal institutions produce positive and negative ecological, economic, political, and sociocultural outcomes of FLR, the endogenous formal institutions have only positive ecological and economic outcomes. On the other hand, endogenous informal institutions produce positive ecological, economic, sociocultural, and political outcomes. This result implies that the involvement of many endogenous institutions in FLR activities both enhances the livelihood of the local people and ensures the sustainability of the intervention while producing limited or no negative outcomes. Hence the need to incorporate many endogenous linked institutions in any FLR intervention in SSA.

**Table 7:** FLR outcomes linked to the typologies of FLR institutions in sub-Saharan Africa (SSA)

Typologies of institutions	*Sub-regions in SSA*	FLR outcomes in SSA								*F* value (*p* value)
		*Ecol*+	*Ecol-*	*Eco*+	*Eco-*	*Pol*+	*Pol-*	*Soc*+	*Soc-*	
Exogenous formal institutions	Western	19	3	14	3	1	6	1	5	5.789*** (0.0005)
	Central	5	0	2	1	3	2	0	0	
	Eastern	17	2	8	1	1	2	1	1	
	Southern	15	10	2	3	1	0	2	1	
	***Total***	**56**	**15**	**26**	**8**	**6**	**10**	**4**	**7**	
Endogenous formal institutions	Western	1	0	5	0	0	0	0	0	4.112*** (0.0042)
	Central	1	0	1	0	0	0	0	0	
	Eastern	7	0	3	0	0	0	0	0	
	Southern	4	0	0	0	0	0	0	0	
	***Total***	**13**	**0**	**9**	**0**	**0**	**0**	**0**	**0**	
Endogenous informal institutions	Western	4	0	7	0	2	0	0	0	10.683*** (0.0000)
	Central	6	0	1	0	0	0	1	0	
	Eastern	7	0	3	0	1	0	0	0	
	Southern	4	0	2	0	0	0	3	0	
	***Total***	**21**	**0**	**13**	**0**	**3**	**0**	**4**	**0**	

[Ecol+ = positive ecological, Ecol- = negative ecological; Eco+= positive economic; Eco- = negative economic; Pol+ = positive political; Pol- = negative political; Soc+= positive sociocultural; Soc- = negative sociocultural; degree of freedom between group = 7; degree of freedom within group = 24]

Concerning the exogenous formal institutions in SSA, the ANOVA results of the study (*F*_(7,24)_ = 5.789, *p* = 0.0005) revealed that a significant difference exists between the FLR outcomes produced by the exogenous formal institutions, of which the positive ecological, economic, negative ecological, economic and political outcomes are the most dominant (Table 7). The positive ecological and economic outcomes were recorded more in the case studies from western and eastern Africa. In contrast, the negative ecological and economic outcomes were recorded more in case studies from southern and western Africa. However, case studies from western Africa recorded the highest negative political outcomes.

From the review, the key positive ecological outcomes associated with exogenous formal institutions include biodiversity restoration and protection, tree-planting, and restoration of forest landscape. For instance, in Burkina Faso, the implementation of the developed forest management plan by the local government authority resulted in forest users groups’ involvement in restoration activities: this resulted in a successful restoration of deforested landscapes; the restored forests persisted for more than nine years (Walters et al. 2021). The review also revealed resistance to planting trees on lands as the main negative ecological outcome of exogenous formal institutions in SSA. For example, a poor land tenure system in Malawi deterred community participation in long-term investments on lands, such as agroforestry (Toth et al. 2019). The main positive economic outcomes linked to exogenous formal institutions include, increased income, increased fodder availability, economic empowerment and provision of alternative livelihood enterprises. For example, the Fandriana-Marolambo landscape restoration program promoted alternative livelihood enterprises such as essential oils extraction, honey production, and small animal and fish farming to improve people’s livelihoods in rural Madagascar (Mansourian et al. 2016).

Additionally, the review revealed systemic market constraints and lack of strategic investment in rural economy as the key negative economic outcomes linked to exogenous formal institutions. For instance, in Malawi, the government’s afforestation program did not prioritize investing in human capital and rural communities’ economies (Whittaker 2020). The review also revealed a lack of transparency and limited powers to local actors as the main negative political outcomes linked to exogenous formal institutions. For example, in Ghana, there is an obscurity and lack of transparency in the management of the plantation forestry fund (Kumeh et al. 2019).

Regarding the endogenous formal institutions in SSA, the ANOVA results of the study (*F*_(7,24)_ = 4.112, *p* = 0.004) showed a significant difference between the FLR outcomes produced by the endogenous formal institutions, of which the positive ecological outcome is the most dominant, followed by the positive economic outcome (Table 7). While the positive ecological outcomes were more dominant in the case studies from eastern and southern Africa, the positive economic outcomes were more dominant in the case studies from western and eastern Africa. From the review, the main positive ecological outcomes linked to endogenous formal institutions are the natural regeneration of forests, tree-planting, and erosion control. For instance, in Uganda, a community-based organization’s involvement in FLR through collaborative forest management arrangements resulted in tree-planting on farms, enrichment tree-planting in the forest, and soil erosion control (Galabuzi et al. 2014). Also, the review revealed income and enhancement in livelihoods as the key positive economic outcomes of endogenous formal institutions. For example, in Kenya, a local environmental management committee regulated the natural regeneration of indigenous plant species (*Suaeda monoica*) that has the potential to generate income (Olukoye and Kinyamario 2009).

For the endogenous informal institutions in SSA, the ANOVA results of the study (*F*_(7,24)_ = 10.683, *p* = 0.0000) showed that there is a significant difference between the FLR outcomes produced by the endogenous informal institutions, of which the positive ecological outcome was the highest, followed by the positive economic, sociocultural, and political outcomes (Table 7). While the positive ecological outcomes were more dominant in the case studies from eastern and central Africa, the positive economic and political outcomes were more dominant in the case studies from western Africa, and the sociocultural outcomes were prevalent in the case studies from southern Africa. The key positive ecological outcomes of endogenous informal institutions in SSA are soil improvement, on-farm tree-planting, conservation, and biodiversity protection. For instance, customary land tenure arrangements in Ghana ensured sustainable land management, which led to soil improvements, on-farm tree-planting and conservation (Asaaga et al. 2020).

Also, endogenous informal institutions’ key positive economic outcomes are access to fuelwood and income. For example, in rural Ethiopia, a local norm of tree-planting provided about 109 tons of fuelwood, about 75% of the total energy demand in the village (Tadele et al. 2020). The main positive political outcome linked to endogenous informal institutions is participation. For instance, in Uganda, for instance, the participation of farmers’ associations in an FLR program embedded the FLR intervention in the local institutional context (Hampson et al. 2017). The key positive sociocultural outcome of endogenous informal institutions is the spiritual connection of the local people to their land, ancestral spirits and God. For example, in the Tshidzivhe, Vuvha, and Thohoyandou communities of South Africa, forest restoration and protection via sacred forests connect local people to their land, ancestors and God (Constant and Taylor 2020).

## Limitations of the Review

This review is restricted to actors and institutions in FLR in SSA. It draws from empirical peer-review articles from English journals. However, there is the likelihood that some salient information in the articles published in other languages, i.e., French, Portuguese, and German journals, could affect the overall results of this study. Hence, future reviews in this field should consider a multi-lingual review of the literature to gain a concrete perspective of the FLR management institutions and actors in SSA. In addition, the study focused on literature from some specified databases like the web of science, Scopus, Google Scholar and Science Direct. However, it is likely that, some relevant information on FLR management institutions and actors in SSA might have been published in regional and national level databases that may not have been captured in the databases that were consulted during this study. A future review should therefore try to consider other regional and local level databases where possible. Moreover, this review focused on the typologies of institutions and their link to FLR outcomes in SSA and the actors’ interests and power manifestations. The study should have also addressed the institutional and actor typologies and their link to the various FLR processes. However, it is impossible to address all this information in a single review paper.

Furthermore, to a larger extent, the review reported separately on actors and institutions in SSA’s FLR. Future studies should present a detailed joint analysis of actors-cum-institutions in SSA’s FLR. Additionally, issues of (in)justice that occurred during SSA’s FLR due to the interests and power manifestation of actors (Elias et al. 2021; Kandel et al. 2021; Kariuki and Birner 2021) were not detailly assessed in this review due to the volume of information captured. Therefore, a future review should pay more attention to issues related to (in)justices in SSA’s FLR. Finally, considering the complex nature of FLR institutions and actors, it is imperative a review pays attention to the methodologies employed in FLR-linked actors and institutional studies. However, the quantum of information in this review makes it impossible to provide a detailed analysis of the methods used in FLR actors-cum-institutional studies in SSA: this, therefore, calls for a review of the methodologies employed in the FLR actors-cum-institutional studies, especially in SSA.

## Synthesis and Conclusions

Forest governance which is about actors’ decision-making and the institutional frameworks that enable the implementation of the decisions (Giessen and Buttoud 2014), is increasingly understood to be the key determining factor to successful forest restoration policy and interventions (Mansourian 2016). The scientific literature has highlighted the importance of actors’ collaboration for achieving the intended effects and political pledges and the significance of institutions, including the dynamics around institutions in different forest landscapes. Yet, a comprehensive understanding of the role played by the different types of formal or informal, endogenous or exogenous institutions, as well as types of actors in shaping FLR and the outcomes they produce, have been missing. This has hindered the definition of an actor-cum-institutions research agenda for FLR, especially in the context of SSA. Yet, in this region, actors from traditional local as well as post-colonial centralized realms shape FLR practices based on their diverse formal and informal interests. Likewise, formal as well as informal institutions stemming from endogenous local or exogenous national or international contexts are known to prevail and collide frequently. Hence, this review addresses the lacunae by drawing from the exogenous and endogenous institutionalism and the ACP lenses to analyze questions linked to (i) FLR actors’ interests and power manifestations; (ii) and the typologies of institutions linked to FLR outcomes in SSA. Based on these objectives, the review identifies future research priorities and questions regarding FLR actors and institutions in SSA. From the systematic literature review of 75 empirical and peer-reviewed journal articles, the following key lessons can be drawn:

First, although actors, both exogenous and endogenous, that shape FLR interventions are interested in the ecological, economic, sociocultural, and political benefits of FLR, while the interest of exogenous actors is skewed more toward ecological FLR benefits, the interest of endogenous actors is more towards economic benefits. This calls for future research to unravel the conditions under which both exogenous and endogenous actors could develop a complimentary view of FLR and its associated benefits. The findings also enable researchers and practitioners to target specific actors in cases where either more economic or ecological impacts are sought for to achieve comprehensive outcomes.

Second, (dis-)incentives and coercion have been the most used power manifestation elements of exogenous actors in SSA’s FLR in modifying the behavior of endogenous actors, to pursue their interests in FLR. Surprisingly, no information in the literature exists about how the endogenous actors of SSA’s FLR manifest power to pursue their interests in FLR. This, therefore, calls for further empirical evidence on FLR actors’ power resources in the context of SSA to uncover how endogenous actors manifest power in the context of FLR, also paying attention to how actors (re)shape institutions to achieve their interests and how institutions (re)shape the behavior of actors. The use of dominant information as a power source also needs to be addressed in the future.

Third, though the review identified that three institutional typologies (i.e., exogenous formal, endogenous informal, and endogenous informal institutions) shape FLR activities in SSA, the exogenous formal institutions were the most dominant thus far observed in the literature. This suggests the so far limited scientific interest in unraveling the traditional and local level structures and processes that shape and decide on the success and failures of FLR activities in SSA. Per the sub-regional distributions, central Africa was the only sub-region that captured more case studies on endogenous informal institutions than exogenous formal institutions. This presupposes that endogenous informal institutions might be very influential in regulating SSA’s FLR activities, albeit unequally across the region. Hence, the need to increase scientific research interests in uncovering the traditional and local structures and processes that regulate the region’s FLR activities.

Finally, although exogenous and endogenous institutions produce ecological, economic, political, and sociocultural FLR outcomes, while the exogenous formal institutions seem to be linked to both negative and positive outcomes, the endogenous formal and informal institutions seem to be linked to only positive outcomes. Therefore, future empirical studies need to consider the conditions under which endogenous institutions (both formal and informal) are likely to produce negative FLR outcomes. Additionally, future studies should empirically identify actors’ compliance levels of the exogenous and endogenous formal and informal typologies of institutions. Future studies should also analyze the effectiveness of FLR-linked institutions towards ensuring successful FLR.

## Supplementary information

Supplementary File 1

Supplementary File 2

## Footnotes

1 Bonn Challenge is the global ambitious commitment to restore about 350 million hectares of degraded forest landscape by 2030 (Bonn Challenge, 2011; UN Climate Summit, 2014).

2 The African Forest Landscape Restoration Initiative (AFR100) is Africa’s commitment to helping achieve the Bonn Challenge (AFR100, 2016). It is a country-led initiative that the African Union introduced to restore about 100 million ha of degraded and deforested landscape in SSA by 2030 (Djenontin et al., 2021).

3 An exogenous formal institution is any structure or process (institution) that emanates from an entity that originates outside the locality of a community of its implementation or influence and has some form of legal recognition by the government.

4 An exogenous informal institution is any structure or process that emanates from an entity outside of the community of its implementation or influence but does not have any official or legal recognition from the government. It could be an unofficial agreement or expectation between exogenous entities and local communities.

5 An endogenous formal institution is any locally crafted structure or process (institution) that has attained legitimacy or recognition by the government.

6 An endogenous informal institution is any of the local levels’ unwritten or uncodified structures and processes transcending generation to generation.

7 By referring to “tree-planting” as a positive ecological outcome, we imply the planting of ecologically and environmentally friendly trees that can lead to the recreation or production of a suitable ecological functionality of the area where the restoration intervention was carried out.

8 “Resistance to reforestation” or “avoiding reforestation” are classified as the negative ecological outcome of FLR interventions because they are the unintended outcomes of FLR interventions that are caused by the institutional framework (such as rules, norms, laws, conventions, etc.) leading to a situation whereby positive ecological outcomes (such as erosion control, carbon sequestration, increase humidity, biodiversity conservation, among others) that would have been achieved if reforestation had succeeded, without resistance, fail to be achieved. This is negative for the ecological functionality of the landscape.

9 Exogenous actors in FLR are the entities that originate from outside the community of the FLR intervention but have interests and the potential to influence the FLR intervention and other actors in the bid to pursue their interests.

10 Endogenous actors in FLR are the local level entities that emanate from the community of the FLR intervention but have interests and the potential to influence the FLR intervention and other actors in the bid to pursue their interests.

## References

[CR1] Acema D, Byakagaba P, Banana AY, Turyahabwe N (2021). Local institutions and the governance of tree resources. Conserv Soc.

[CR2] AFR100 (2016) African Forest Landscape Restoration Initiative. http://www.afr100.org/sites/default/files/AFR100%20Overview_ENG.pdf (Accessed 12 Apr 2022)

[CR3] Agúndez D, Lawali S, Mahamane A, Alía R, Soliño M (2020). Farmers’ preferences for conservation and breeding programs of forestry food resources in Niger. Forests.

[CR4] Artmann M, Sartison K (2018). The role of urban agriculture as a nature-based solution: a review for developing a systemic assessment framework. Sustainability.

[CR5] Asaaga FA, Hirons MA, Malhi Y (2020). Questioning the link between tenure security and sustainable land management in cocoa landscapes in Ghana. World Dev.

[CR6] Ashley R, Russell D, Swallow B (2006). The policy terrain in protected area landscapes: challenges for agroforestry in integrated landscape conservation. Biodivers Conserv.

[CR7] Baruah M, Bobtoya S, Mbile P, Walters G (2016). Governance of restoration and institutions: working with Ghana’s community resource management areas. World Dev Perspect.

[CR8] Benjamin EO, Ola O, Sauer J, Buchenrieder G (2021). Interaction between agroforestry and women’s land tenure security in sub-Saharan Africa: a matrilocal perspective. For Policy Econ.

[CR9] Bonn Challenge (2011) A global effort. https://www.bonnchallenge.org/content/challenge (Accessed 17 Jun 2022)

[CR10] Carter J, Schmidt P, Robinson T, Stadtmüller T, Nizami A (2009). Forests, landscapes and governance: multiple actors, multiple roles.

[CR11] Clay L, Hay-Smith EJC, Treharne GJ, Milosavljevic S (2015). Unrealistic optimism, fatalism, and risk-taking in New Zealand farmers’ descriptions of quad-bike incidents: a directed qualitative content analysis. J Agromed.

[CR12] Constant NL, Taylor PJ (2020). Restoring the forest revives our culture: ecosystem services and values for ecological restoration across the rural-urban nexus in South Africa. For Policy Econ.

[CR13] Djenontin IN, Zulu LC, Etongo D (2021). Ultimately, what is forest landscape restoration in practice? Embodiments in sub-Saharan Africa and implications for future design. Environ Manag.

[CR14] Elias M, Joshi D, Meinzen-Dick R (2021). Restoration for whom, by whom? A feminist political ecology of restoration. Ecol Restor.

[CR15] FAO (2020) *Global Forest Resources Assessment 2020: Main report*. https://www.fao.org/3/ca9825en/ca9825en.pdf (Accessed 14 Apr 2022)

[CR16] Fleetwood S (2008). Structure, institution, agency, habit and reflexive deliberation. J Inst Econ.

[CR17] Folefack AJJ, Darr D (2021). Promoting cocoa agroforestry under conditions of separated ownership of land and trees: strengthening customary tenure institutions in Cameroon. Land Use Policy.

[CR18] Foundjem-Tita D, Degrande A, Kamdem CB (2021). National and International Policies and Policy Instruments in the Development of Agroforestry in Chad. Sustainability.

[CR19] Gakou-Kakeu J, Di Gregorio M, Paavola J, Sonwa DJ (2022). REDD+ policy implementation and institutional interplay: evidence from three pilot projects in Cameroon. For Policy Econ.

[CR20] Galabuzi C, Eilu G, Mulugo L, Kakudidi E, Tabuti JRS, Sibelet N (2014). Strategies for empowering the local people to participate in forest restoration. Agrofor Syst.

[CR21] Giessen L, Buttoud G (2014). Assessing forest governance-analytical concepts and their application. For Policy Econ.

[CR22] Guariguata M, Brancalion P (2014). Current challenges and perspectives for governing forest restoration. Forests.

[CR23] Hampson K, Leclair M, Gebru A, Nakabugo L, Huggins C (2017). “There is No Program Without Farmers”: interactive radio for forest landscape restoration in Mount Elgon Region, Uganda. Soc Nat Resour.

[CR24] Hsieh H-F, Shannon SE (2005). Three approaches to qualitative content analysis. Qual Health Res.

[CR25] IUCN, WWF (2000) *Forests reborn: a workshop on forest Restoration [overview]*. https://www.iucn.org/sites/dev/files/import/downloads/flr_segovia.pdf (Accessed 16 May 2022)

[CR26] Kandel M, Agaba G, Alare RS, Addoah T, Schreckenberg K (2021). Assessing social equity in farmer-managed natural regeneration (fmnr) interventions: findings from Ghana. Ecol Restor.

[CR27] Kariuki J, Birner R (2021). Exploring gender equity in ecological restoration: the case of a market-based program in Kenya. Ecol Restor.

[CR28] Kimengsi JN, Mukong AK, Giessen L, Pretzsch J (2022). Institutional dynamics and forest use practices in the Santchou Landscape of Cameroon. Environ Sci Policy.

[CR29] Kimengsi JN, Owusu R, Djenontin IN, Pretzsch J, Giessen L, Buchenrieder G, Acosta AN (2022). What do we (not) know on forest management institutions in sub-Saharan Africa? A regional comparative review. Land Use Policy.

[CR30] Kimengsi JN, Mukong AK (2022) Forest resource endogenous cultural institutions in rural Cameroon: compliance determinants and policy implications. J Environ Plan Manag, 1–22. 10.1080/09640568.2022.2034606

[CR31] Kimengsi JN, Abam CE, Forje GW (2021) Spatio-temporal analysis of the ‘last vestiges’ of endogenous cultural institutions: implications for Cameroon’s protected areas. GeoJournal, 1–18. 10.1007/s10708-021-10517-z

[CR32] Kiptot E, Franzel S (2012). Gender and agroforestry in Africa: a review of women’s participation. Agrofor Syst.

[CR33] Krott M, Bader A, Schusser C, Devkota R, Maryudi A, Giessen L, Aurenhammer H (2014). Actor-centred power: the driving force in decentralised community based forest governance. For Policy Econ.

[CR34] Kumeh EM, Kyereh B, Oduro KA, Brobbey LK, Nketiah SK (2019). Transparency in the governance of landscape restoration finance: a case study of Ghana’s Forest Plantation Development Fund. Sci Afr.

[CR35] Laestadius L, Buckingham K, Maginnis S, Saint-Laurent C (2015). Before Bonn and beyond: the history and future of forest landscape restoration. Unasylva.

[CR36] Mansourian S (2016). Understanding the relationship between governance and forest landscape restoration. Conserv Soc.

[CR37] Mansourian S (2017). Governance and forest landscape restoration: a framework to support decision-making. J Nat Conserv.

[CR38] Mansourian S, Razafimahatratra A, Ranjatson P, Rambeloarisao G (2016). Novel governance for forest landscape restoration in Fandriana Marolambo, Madagascar. World Dev Perspect.

[CR39] Mansourian S, Berrahmouni N (2021) Review of forest and landscape restoration in Africa. Accra: file:///Users/Raphael/Downloads/Review of Forest and Landscape Restoration in Africa_EN.pdf&hl=en. (Accessed 17 May 2022)

[CR40] Mbile PN, Atangana A, Mbenda R (2019). Women and landscape restoration: a preliminary assessment of women-led restoration activities in Cameroon. Environ Dev Sustain.

[CR41] North D (1990). Institutions, institutional change and economic performance.

[CR42] Olukoye G, Kinyamario J (2009). Community participation in the rehabilitation of a sand dune environment in Kenya. Land Degrad Dev.

[CR43] Osei-Tutu P, Pregernig M, Pokorny B (2015). Interactions between formal and informal institutions in community, private and state forest contexts in Ghana. For Policy Econ.

[CR44] Ostrom E (1990). Governing the commons: The evolution of institutions for collective action.

[CR45] Ostrom E (1992). Crafting institutions for self-governing irrigation systems.

[CR46] Palmer CG, Fry A, Libala N, Ralekhetla M, Mtati N, Weaver M, Scherman P-A (2022). Engaging society and building participatory governance in a rural landscape restoration context. Anthropocene.

[CR47] Petticrew M, Robert H (2006) Why do we need systematic. In *Systematic reviews in the social sciences: a practical guide* (pp. 1–27). Oxford, UK: Blackwell Publishing Ltd

[CR48] Pramova E, Locatelli B, Djoudi H, Somorin OA (2012). Forests and trees for social adaptation to climate variability and change. Wiley Interdiscip Rev: Clim Change.

[CR49] Reij C, Pasiecznik N, Mahamoudou S, Kassa H, Winterbottom R, Livingstone J (2020) Dryland restoration successes in the Sahel and Greater Horn of Africa show how to increase scale and impact. In Pasiecznik N, Reij C (Eds.), *Restoring African Drylands* (Vol. 60, pp. 1–24). Bogor, Indonesia: The Center for International Forestry Research (CIFOR)

[CR50] Sanginga PC, Kamugisha RN, Martin AM (2010). Strengthening social capital for adaptive governance of natural resources: A participatory learning and action research for bylaws reforms in Uganda. Soc Nat Resour.

[CR51] Sanou L, Savadogo P, Ezebilo EE, Thiombiano A (2019). Drivers of farmers’ decisions to adopt agroforestry: evidence from the Sudanian savanna zone, Burkina Faso. Renew Agric Food Syst.

[CR52] Schusser C, Krott M, Movuh MCY, Logmani J, Devkota RR, Maryudi A, Bach ND (2015). Powerful stakeholders as drivers of community forestry—results of an international study. For Policy Econ.

[CR53] Schusser C, Krott M, Movuh MCY, Logmani J, Devkota RR, Maryudi A, Salla M (2016). Comparing community forestry actors in cameroon, indonesia, namibia, nepal and germany. For Policy Econ.

[CR54] Shvetsova O (2003). Endogenous selection of institutions and their exogenous effects. Const Political Econ.

[CR55] Stanturf JA (2021). Forest landscape restoration: building on the past for future success. Restor Ecol.

[CR56] Stanturf JA, Mansourian S (2020). Forest landscape restoration: state of play. R Soc open Sci.

[CR57] Stanturf JA, Kant P, Lillesø J-PB, Mansourian S, Kleine M, Graudal L, Madsen P (2015). Forest landscape restoration as a key component of climate change mitigation and adaptation (Vol. 34).

[CR58] Tadele M, Birhane E, Kidu G, G-Wahid H, Rannestad MM (2020). Contribution of parkland agroforestry in meeting fuel wood demand in the dry lands of Tigray, Ethiopia. J Sustain For.

[CR59] Toth GG, Nair P, Duffy CP, Franzel SC (2017). Constraints to the adoption of fodder tree technology in Malawi. Sustain Sci.

[CR60] Toth GG, Nair PR, Jacobson M, Widyaningsih Y, Duffy CP (2019). Malawi’s energy needs and agroforestry: Impact of woodlots on fuelwood sales. Energy Sustain Dev.

[CR61] Turyahabwe N, Geldenhuys C, Watts S, Obua J (2007). Local organisations and decentralised forest management in Uganda: roles, challenges and policy implications. Int For Rev.

[CR62] UN Climate Summit (2014) *New York Declaration on forests: declaration and action agenda*. https://www1.undp.org/content/dam/undp/library/Environment%20and%20Energy/Forests/New%20York%20Declaration%20on%20Forests_DAA.pdf (Accessed 12 Apr 2022)

[CR63] Vallino E (2014). The tragedy of the park: an agent-based model of endogenous and exogenous institutions for forest management. Ecol Soc.

[CR64] Walters G, Baruah M, Karambiri M, Adjei PO-W, Samb C, Barrow E (2021). The power of choice: How institutional selection influences restoration success in Africa. Land Use Policy.

[CR65] Whittaker AR (2020). Why we fail: stakeholders’ perceptions of the social and ecological barriers to reforestation in southern Malawi. People Nat.

[CR66] Wibowo A, Giessen L (2015). Absolute and relative power gains among state agencies in forest-related land use politics: The Ministry of Forestry and its competitors in the REDD+ Programme and the One Map Policy in Indonesia. Land Use Policy.

[CR67] Yeboah-Assiamah E, Muller K, Domfeh KA (2017). Institutional assessment in natural resource governance: a conceptual overview. For Policy Econ.

[CR68] Yeboah-Assiamah E, Muller K, Domfeh KA (2019). Two sides of the same coin: formal and informal institutional synergy in a case study of wildlife governance in Ghana. Soc Nat Resour.

